# Responsiveness to City Service Requests, Life Satisfaction, and Horizontal Inequality: Does Good Local Governance Improve Subjective Well-Being for All?

**DOI:** 10.3390/ijerph23010132

**Published:** 2026-01-21

**Authors:** Danyel P. L. Tharakan, Tiffany N. Ford

**Affiliations:** School of Public Health, University of Illinois Chicago, Chicago, IL 60612, USA; dthara2@uic.edu

**Keywords:** neighborhood inequality, geographic information system, subjective well-being, governance

## Abstract

**Highlights:**

**Public health relevance—How does this work relate to a public health issue?**

- This paper examines the effects of local governance on life satisfaction, an important indicator of overall health and wellness.
- It also examines whether neighborhood-level race and ethnicity moderate this relationship.

**Public health significance—Why is this work of significance to public health?**

- Our findings show that good local governance significantly improves overall life satisfaction.
- We also found that good local governance improves life satisfaction more in white neighborhoods than in Black and Hispanic neighborhoods.

**Public health implications—What are the key implications or messages for practitioners, policy makers, and/or researchers in public health?**

- These findings indicate that meeting residents’ basic needs for public services is not sufficient to improve life satisfaction in Black and Hispanic neighborhoods.
- More actively democratic processes of co-governance may be necessary to improve life satisfaction in Black and Hispanic neighborhoods.

**Abstract:**

Local governance has been found to be an important determinant of individuals’ subjective well-being (SWB) in cross-municipality studies in Europe and Asia. In addition, previous literature suggests that increasing access to determinants of SWB provides lesser SWB benefit to racial minorities compared to white people in the United States (U.S.). Given this context, we ask the following: (1) does good local governance improve SWB in the U.S.? and (2) does good local governance improve SWB for Black and Hispanic people equally compared to white people? To answer these questions, we examine Chicago, Illinois, the third-largest city in the U.S. with substantial Black and Hispanic populations. We model local governance, our independent variable, as the number of weeks for the municipality to respond to pothole service requests reported to the city’s non-emergency services system. Our dependent variable was life satisfaction, measured by the Cantril Ladder. Covariates included self-reported health problems, lack of money for food, sex, age, age-squared, and marital status. Neighborhood race/ethnicity was tested as a moderator of the primary relationships. We estimated linear regression models with and without race × governance interactions. Our findings demonstrate that local governance is an important determinant of SWB, but that it benefits SWB in white neighborhoods more than in Black/Hispanic neighborhoods.

## 1. Introduction

### 1.1. Subjective Well-Being

Subjective well-being (SWB) is a term that covers a variety of self-reported measures of an individual’s quality of life (QoL), including perceptions of life satisfaction, optimism, stress, and beyond. SWB variables are often measured on numeric scales, such as the validated Cantril Self-Anchoring Striving Scale (more commonly known as the Cantril Ladder), which asks respondents to imagine where their actual life stands relative to their worst and best possible lives [1,2]. SWB can be impacted by most conditions of life, such as age, race, gender, and income [3]. Conversely, greater SWB has been found to be associated with important aspects of life such as social relationships, residential location, economic and political participation, and general health [1,4]. SWB can be divided into three domains: eudaimonic, measured as purpose in life; hedonic, measured as happiness and stress; and evaluative, measured as optimism and life satisfaction, among others [5]. For the purposes of this study, we focus on life satisfaction, as it is considered one of the more stable measures of SWB and is therefore more frequently used in policymaking [6].

### 1.2. Quality of Governance

More recent research has focused on the connection between quality of governance (QoG) and SWB [7]. Measures for QoG vary at different levels of government—from municipal to international. The World Bank Worldwide Governance Indicator Project includes six indicators grouped into three dimensions [8]:

1. The process by which governments are elected, monitored, and replaced:
  - Voice and accountability
  - Political stability and absence of violence
2. The capacity of governance to effectively formulate and implement sound policies:
  - Government effectiveness
  - Regulatory quality
3. The respect of citizens and the state for the institutions that govern economic and social interactions among them:
  - Rule of law
  - Control of corruption

For the purposes of evaluating QoG at the municipal level, voice and accountability, government effectiveness, and control of corruption have been found to be the most relevant [7]. Previous studies have found a strong and significant positive relationship between QoG and SWB at the cross-national level as well as at the municipal level in Europe [7,9,10]. As such, we consider QoG to be one of the determinants of SWB.

### 1.3. Measuring Quality of Governance

For the purposes of this study, we will be approximating two indicators of QoG—government effectiveness and voice and accountability—using the municipal government’s responsiveness to potholes reported by residents. Specifically, QoG is measured as the number of weeks it takes for potholes reported to the city’s non-emergency city services system (311 system) to be reported as filled. Most potholes are filled by the City of Chicago Department of Transportation (CDOT); some are filled due to routine maintenance, while others are filled due to resident complaints or political priorities.

We decided to use pothole servicing time as a proxy for QoG after a thorough review of available data, the literature on governance, transportation, and QoL, government publications, and news media. Some of the most widely available data directly quantifying government responsiveness to residents’ concerns comes from 311 systems, which in the U.S. are typically unified communication systems which allow residents to request services from their municipal government [11]. In Chicago, Illinois, our area of study, potholes were the third most prevalent category of request (over 247,000 service requests), after only aircraft noise complaints and graffiti removal [12]. Due to limitations in the dataset elaborated in the Limitations Section, potholes presented the best category of 311 data to evaluate.

In addition to the data limitations, the literature, government publications, and news media suggest that potholes have a salient impact on residents’ QoL. Papers from as far afield as Sweden, Japan, and Ireland have found that improved roadway quality has a significant positive impact on quality of life [13,14,15]. A report from the Minnesota Department of Transportation found significant relationships between multiple transportation performance measures, including roadway surface quality, and QoL [16]. A report prepared for the Illinois Chamber of Commerce found that damage from potholes cost the average Chicago driver USD 633 annually in added vehicle maintenance costs [17]. Potholes and poor road quality are of high concern to residents, as highlighted by endless local news reports [18,19,20].

Finally, and significantly for the purposes of evaluating racial discrepancies in QoG, QoL, and the relationship between them, there is evidence that Black and Hispanic neighborhoods in Chicago receive poorer quality services. Chicago’s 50 municipal elected officials (alders) receive a discretionary budget of USD 1.5 million annually to spend on projects in their wards, the majority of which is spent on roadway maintenance [21]. However, a report from the Chicago Inspector General found large inequities in this funding model as it does not account for the actual infrastructure needs in each ward. After accounting for needs, seven of the ten least-funded wards were majority Black and Hispanic on the predominantly lower-income far-south side of the city, while all ten of the most-funded were plurality white wards on the higher-income north side of the city [21].

Based on this information and data limitations, we believe that the time to fill potholes reported to 311 is a good approximator of government effectiveness and voice and accountability for the purposes of evaluating the effect of QoG on QoL and whether this relationship is moderated by race [8].

### 1.4. Significance

As of this writing, the authors could not find any research examining the link between governance and SWB at the state or local level in the U.S. Previous studies have examined the link at the international level as well as the domestic and municipal levels in Europe [7,9,22]. The U.S. presents a different sociopolitical context that may impact this relationship. Firstly, institutional trust has been found to mediate the role between QoG and SWB, and the U.S. has the third-lowest level of government trust out of the 38 members of the Organization for Economic Cooperation and Development (OECD) [23,24]. Secondly and most central to this paper, the U.S. presents an entirely different racial and ethnic context that has been shown to have significant impacts on SWB [3]. Both an individual’s race/ethnicity and the racial/ethnic demographics of the neighborhood in which they live have been found to play an important role in health outcomes [25,26,27], yet there have been comparatively few studies on the relationship between neighborhood race/ethnicity and SWB. Recent literature has found evidence of the Minorities’ Diminished Returns (MDR) theory for SWB, i.e., that increasing socioeconomic status (SES) has lesser effects on SWB for Black and Hispanic people than for white people in the U.S. [28]. As we consider governance to be another determinant of SWB akin to SES, we are investigating whether the MDR theory may hold for governance and SWB as well.

### 1.5. Purpose of the Present Study

In this study, we examine the link between good governance, measured as the number of weeks for reported potholes to be filled, and SWB, measured as life satisfaction on a scale of 0–10. This represents our first question: does good local governance improve SWB? Secondly, we examine if the link between good governance and SWB is moderated by the racial demographics of the geographic area of analysis. This represents our second question: does good local governance improve SWB for all? Or, instead, do we see evidence of the MDR theory holding for governance and SWB?

## 2. Materials and Methods

### 2.1. Study Area

We use Chicago in our study for several reasons. First, Chicago, Illinois, is the third-largest city in the US and was home to over 790,000 Hispanic people and nearly 830,000 non-Hispanic Black people in 2016, the end year of our time period of analysis—29.1% and 30.6% of the city’s population, respectively—ensuring a sufficient sample size in our demographic variable of interest [29]. Second, Chicago has a well-developed and highly utilized 311 system, which enables residents to submit service requests. The 311 system is a public service which residents can use to request city services online, through a mobile application, or by calling 311 [30]. The system was implemented in 1999 to make it easier for residents to access municipal services by calling one 3-digit number to access all services, as opposed to the previous system, which required residents to dial separate 10-digit numbers for individual city departments for different service requests [31]. Chicago’s 311 system now handles between three and four million service requests annually, including requests involving animal control, consumer and employee protection, garbage pickup, health violations, building safety, infrastructure repairs, and beyond [30]. Third, pothole servicing has been a major political talking point for decades in Chicago [32]. Unserviced potholes cost the average Chicagoan USD 633 annually in added vehicle operating and repair costs [17]. Given the high salience of potholes in Chicago, pothole servicing should increase the quality of life of local residents.

### 2.2. Study Variables

The Gallup Daily United States Poll (Gallup Daily) was a nationally representative survey focused on the health and SWB of U.S. adults that ran from 2008 to 2017 and interviewed between 500 and 1000 U.S. adults per day using random-digit-dial cellphone and landline numbers [33]. The dependent variable in this study was individual-level life satisfaction (N = 5173), measured using the Self-Anchoring Striving Scale, also referred to as the Cantril Ladder [2], a commonly used and validated measure [34,35,36]. The Cantril Ladder measures life satisfaction on a scale from 0 (worst possible life) to 10 (best possible life). These data came from the 2012–2016 Gallup Daily. Gallup Daily is a proprietary dataset that we have permission to use in this study because of the senior author’s (TF) joint research with Carol Graham, a Senior Scientist at Gallup Inc. 2016 is the latest year of Gallup Daily data that TF has access to. Despite the age of this data, due to the relative stability of life satisfaction and the near-constant political, social, and economic salience of potholes, we believe our analysis still offers valuable insights into the relationship between QoG and SWB [6,13,14,15,16,17,18,19,20,21,32]. The smallest geographic unit of analysis available for respondents was their postal (ZIP) code. ZIP codes, unlike census tracts or block groups, are not geographically sized to include relatively similar population sizes. There are 58 ZIP code geographies in Chicago, ranging in area from just 0.25 square kilometers in the city center to 36 square kilometers in outlying areas [37].

We spatially linked individual data on life satisfaction with publicly available data on potholes from the City of Chicago 311 system. We examined pothole servicing using data obtained from the City of Chicago, Illinois, 311 system between 2012 and 2016. Chicago’s 311 system is a non-emergency city service telephone line that compiles service requests from city residents. When a resident calls 311 to report a pothole in Chicago, a service request is created, which is automatically routed to CDOT for their action. Callers may track the status of their service requests and receive available updates [30]. The system is widely used, with over 247,000 pothole service requests from 2012 to 2016 [12]. CDOT is also highly responsive to these requests, with over 86% of potholes reported to 311 filled [12]. Pothole servicing was measured by calculating the median weeks it took CDOT to fill 311-reported potholes in each ZIP code from 2012 to 2016.

Following previous literature on the relationship between QoG and SWB from Spain and Italy, we include individual-level controls including demographic variables (age, age-squared, gender, and marital status) and quality-of-life variables (self-reported health problems and not having enough money for food) from the Gallup Daily United States Poll respondents [7,24]. We also controlled for the total number of service requests in each ZIP code, as previous literature suggests that Black and Hispanic people may be less likely to request city services even in areas with need [11]. Geographic-level demographic data at the ZIP code tabulation area (ZCTA) level included racial demographics, specifically the percentages of the population that are non-Hispanic white, non-Hispanic Black, and Hispanic or Latino from the U.S. Census Bureau American Community Survey (ACS) 5-year estimates (2012–2016). The geography for the SWB data and pothole data is ZIP code, while that of the neighborhood demographic data is ZCTA. ZIP codes and ZCTAs in Chicago are very similar geographies but differ slightly. Due to the limited geographic information available in the SWB data, this slight mismatch is a necessary limitation in ZIP/ZCTA-level analyses [38].

### 2.3. Analysis

**Figure A1:**
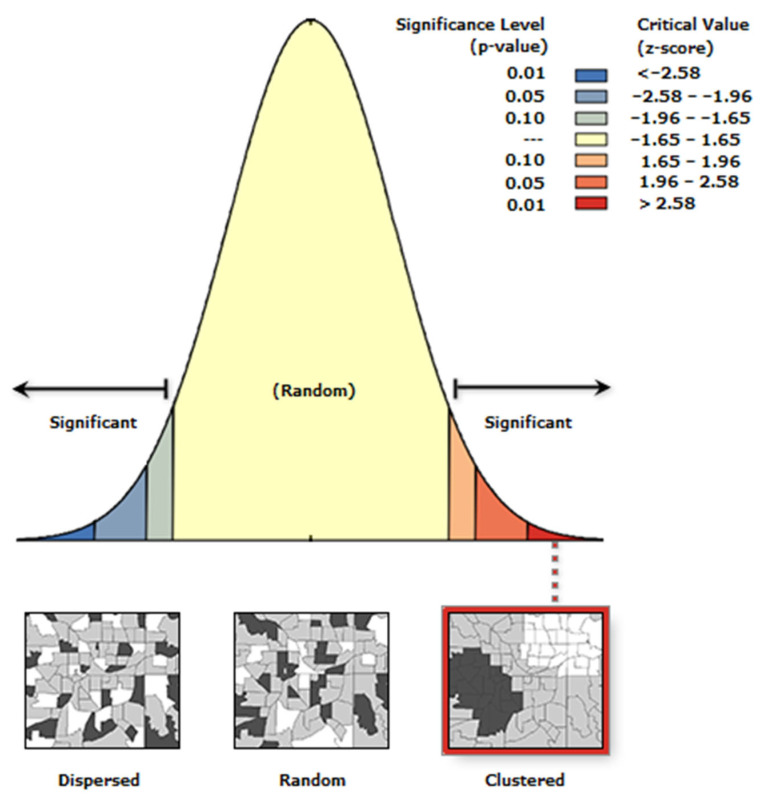
Global Moran’s I explanatory image.

We linked the ZIP code-level 311 and ACS data to individuals in Gallup and mapped our main study variables using ArcGIS Pro (v3.0.3, ESRI, Inc., Redlands, CA, USA) to assess the spatial distribution of life satisfaction (the outcome), time to completion of a service request (primary independent variable), and potential covariates in the study area. Since governance is likely to be similar between nearby neighborhoods—for example, an entire portion of a street could be repaved within a single ZIP code—we tested for global spatial clustering with Moran’s I in GIS to confirm our later use of clustered statistical analysis techniques [39]. The Moran’s I value indicated significant positive spatial autocorrelation for both SWB and the time to fill potholes (Appendix A; Table A1 and Figure A1).

All statistical analyses were conducted in Stata, version 18.5. We first used univariate analyses to describe the characteristics of the study population. Variables are presented as mean, standard deviation, and minimum and maximum value. To assess the association between living in an area with good governance and life satisfaction, we fit three linear regression models of generalized estimating equations (GEE) with an identity link function and exchangeable correlation matrix. Model 1 estimated the association between life satisfaction and time to completion of a service request after adjusting for the number of service requests in each ZIP code. Model 2 added possible individual-level sociodemographic confounders to our model—sex/gender, age in years, age-squared, marital status, and race/ethnicity. Model 3, which we consider our main specification, included potential confounding in two domains that are associated with life satisfaction—an individual’s health status and financial standing. Standardized beta coefficients and ZIP code-clustered robust standard errors were calculated and reported for each model [26]. In addition to the population-averaged GEE models, we performed random intercept multi-level models with the same sets of covariates as sensitivity analyses.

To assess if the association between living in an area with good governance and life satisfaction varied based on the race/ethnicity of an area, we relied on our main specification but included two ZIP code-level race/ethnicity × time to completion of service request interaction terms in two separate models. In our first moderation model, we considered whether the association varied based on the absolute race/ethnicity of an area by including a percent Black and Hispanic of an area × time to completion of service request interaction term. Second, we assessed whether the association varied based on the relative race/ethnicity by including a binary variable coded as 1 if the percent Black and Hispanic of an area was above the city average and 0 otherwise; this variable interacted with time to completion of service request. Finally, we perform a Wald test to assess the significance of each interaction. We also performed a sensitivity analysis examining the interaction between individual, rather than neighborhood, race/ethnicity × time to completion of service request.

We evaluated fit for all GEE models by calculating and reporting Zheng’s marginal R-squared (R^2^_marg_), which estimates the proportion of the variance in life satisfaction that is explained by the independent variables in each model [40]. R^2^_marg_ equals the squared value of the correlation between the observed and predicted life satisfaction and shares the same interpretation as R^2^ in an ordinary least squares regression. Statistical significance was assessed at *p* < 0.05.

## 3. Results

### 3.1. Descriptive Statistics and Geographic Distribution

Table 1 shows the summary statistics of the main variables. The life satisfaction, female, age, age-squared, married, race, not enough money for food, and health problems variables are reported at the individual survey respondent level (N = 5173). The weeks to complete service request and service requests per ZIP code variables are averaged across the ZIP code level (N = 58). At the individual level, life satisfaction ranged from 0 to 10, with a mean of 7.01. Just over 50% of respondents were female, the mean age of respondents was just under 43, and 42% were married. The majority of respondents were non-Hispanic white (53.0%), followed by non-Hispanic Black (31.7%) and Hispanic (15.3%). In total, 16% report not having enough money for food, while 18% report having health problems. At the ZIP code level, the median weeks to complete a service request ranged from 0.14 (1 day) to 1.71 (12 days), with an average of 0.89 (6.2 days). The number of service requests per ZIP code ranged from 337 to 9340, with an average of 4605.

**Figure 1:**
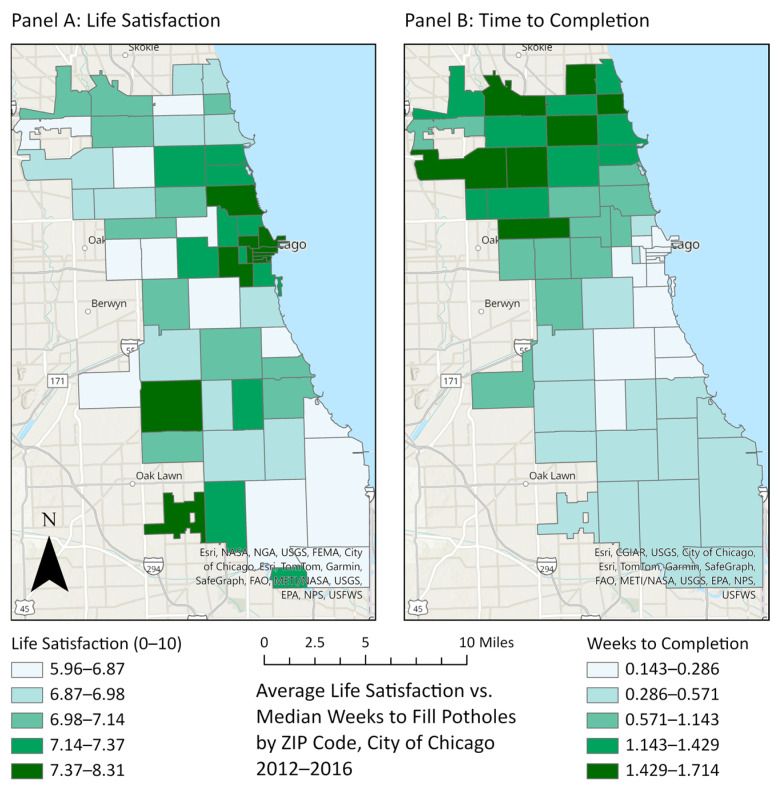
Life satisfaction and weeks to complete pothole service request by ZIP code, Chicago, Illinois, USA, 2012–2016.

Figure 1 shows the average life satisfaction (Panel A) and the median number of weeks for a pothole reported to 311 to be filled (Panel B) for each of the 58 ZIP codes in Chicago. The map colors are grouped by quantiles. As shown in Panel A, the average life satisfaction ranges from 5.96 to 8.31. The ZIP codes in the highest quantile of life satisfaction are mostly grouped around the central business district (the “Loop”) and the areas immediately to the north and west, including the affluent and majority-white neighborhoods of West Loop, Streeterville, Lincoln Park, and Gold Coast. By contrast, ZIP codes in the lowest quantile are mostly in the far-west and southeast portions of the city, including the lower-income, majority-Black neighborhoods of Austin, Garfield Park, South Shore, and South Chicago. Panel B shows that CDOT responds to pothole complaints relatively quickly: the median weeks to completion ranges from 0.143 to 1.714 (about 1–12 days). ZIP codes in the lowest quantile (indicating fastest time to completion) are once again centered around the Loop and to the near north and west, but also extend to the near south to include the racially mixed and middle-upper-income neighborhoods of South Loop, Douglas, Kenwood, and Hyde Park. ZIP codes in the highest quantile are mostly to the far-north and northwest, including racially mixed and middle-income neighborhoods such as Portage Park, Albany Park, and Rogers Park.

### 3.2. Does Good Governance Improve SWB?

**Figure 2:**
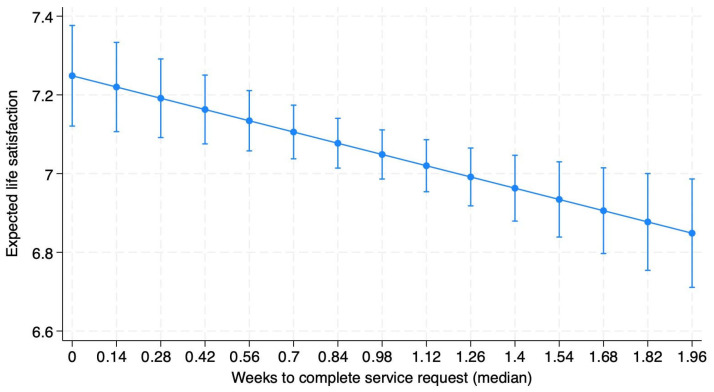
Expected life satisfaction versus weeks to complete service request using Model 1.3 specifications.

Table 2 shows the three population-average GEE models used in our analysis. All models included weeks to completion as the independent variable and life satisfaction as the dependent variable. Model 1.1 only controlled for the number of service requests in a given ZIP code and found that weeks to completion has a negative correlation with life satisfaction that is significant at the 5% level. Model 1.2 added controls for respondents’ demographic characteristics that may impact their life satisfaction, including gender, age, age-squared, marital status, and race. This model increased the significance of weeks to completion’s correlation to the 0.1% level, and it had a higher correlation and more significant relationship with life satisfaction than a respondent’s gender or race. Finally, Model 1.3 added controls for life situations that may impact life satisfaction, including self-reporting health problems and not having enough money for food. Weeks to completion remained significant at the 0.1% level in this model, with a magnitude on par with gender and about half as impactful as having self-reported health problems and a third as impactful as reporting not having enough money for food. As shown in Figure 2, using Model 1.3 specifications, a one-week increase in the average time to fill potholes at the ZIP code level results in a 0.2 decrease in individuals’ life satisfaction in that ZIP code. Sensitivity analyses using random intercept multi-level models had very similar results, validating our population-averaged GEE analysis, but are not reported here.

### 3.3. The Importance of Neighborhood Effects: Does Good Governance Improve SWB for All?

Table 3 presents the main effect of racial neighborhood differences on life satisfaction and its interaction with time to completion of a service request after adjusting for covariates. Only key variables are presented in Table 3 for simplicity. In Model 2.1, the main effect of a higher percent Black/Hispanic population is not significant. In Model 2.2 a higher percent Black/Hispanic population is associated with slightly lower life satisfaction. Also in Model 2.2, the percent Black/Hispanic population × weeks to completion interaction is statistically significant, though the coefficient is very small. This suggests that in ZIP codes with a higher percentage of Black/Hispanic residents, the negative effect of longer service times on life satisfaction is at least slightly less severe. Model 2.3 shows that there is no significant difference in life satisfaction between above-average and below-average Black/Hispanic ZIP codes when not considering interaction. In ZIP codes with above-average Black/Hispanic populations, the negative impact of longer service times on life satisfaction is significantly moderated (Model 2.4). Put another way, the detrimental effect of delays in service completion is less pronounced in these above-average Black/Hispanic neighborhoods.

**Figure 3:**
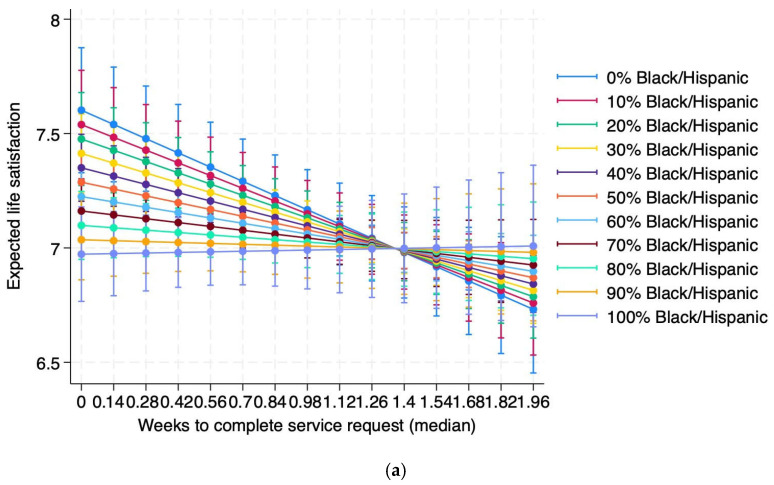

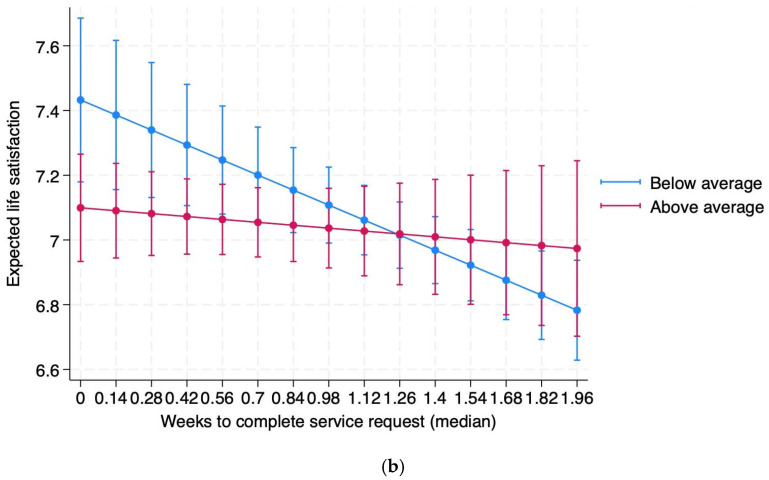
Expected life satisfaction vs. weeks to complete service request by (**a**) absolute ZIP code race/ethnicity (Model 2.2) and (**b**) relative ZIP code race/ethnicity (Model 2.4).

Figure 3 more clearly illustrates the moderating effect of race/ethnicity on the relationship between service time and life satisfaction. Figure 3a shows the results from Model 2.2, which uses absolute percentage of the Black/Hispanic population. As shown, the negative relationship between the two variables weakens as the Black/Hispanic percentage of the population increases to become nearly no relationship above 70%. Figure 3b shows the results from Model 2.4, which instead uses only two classes of neighborhood—ones with Black/Hispanic population above or below the citywide average. The relationship for below-average Black/Hispanic neighborhoods (i.e., whiter neighborhoods) is negative, while the relationship for above-average Black/Hispanic is close to zero. Therefore, modeling in both absolute percentage terms and relative terms, we find that the relationship between good local governance and life satisfaction is weaker in neighborhoods with a higher proportion of Black and Hispanic people. Sensitivity analyses examining the interaction effect of individual race/ethnicity × weeks to completion were not statistically significant.

## 4. Discussion

This study examined two fundamental questions: (1) if good local governance improves life satisfaction in the U.S., and, most novelly, (2) if good local governance improves life satisfaction equally for all, examining the case of Chicago, IL, U.S. from 2012 to 2016. In response to question (1), we find that good local governance, modeled as the number of weeks for the local government to respond to pothole service requests, has a significant, positive effect on life satisfaction. We find this effect both with and without individual demographic and quality-of-life control variables. We found that the magnitude of impact of good governance on life satisfaction was equivalent to that of gender, one-half that of having health problems, and one-third that of not having enough money for food (Table 2). Considering how impactful these serious issues are on SWB, it is notable that good governance is even on the same order of magnitude [41,42].

In terms of absolute impact, we find that a one-week increase in the time it takes for the local government to respond to a pothole service request results in a decrease of 0.20 in life satisfaction (Figure 2). Our findings are in line with previous research at the cross-national level as well as research at the municipal level in Spain that found that good governance at the national and municipal level is significantly and positively associated with SWB [7,9,10]. The findings are also in line with previous studies from Sweden, Japan, and Ireland finding a positive relationship between roadway quality and QoL [13,14,15]. This also logically makes sense, as potholes cost the average Chicago driver USD 633 annually in added vehicle maintenance costs, and fixing the roads is a constant spending priority of politicians locally in Chicago and statewide in Illinois [17,21].

In the case of question (2), we find that good governance does not improve life satisfaction for all equally. Rather, neighborhood race/ethnicity has a significant moderating effect on the relationship between good local governance and life satisfaction. We specifically found that in neighborhoods with a higher Black and Hispanic population, the relationship between good local governance and life satisfaction is weaker. This finding holds both while modeling the Black and Hispanic population in absolute-percentage terms (Figure 3a) as well as relative to the average for each ZIP code (Figure 3b). In other words, better local governance does not translate into higher levels of life satisfaction in Black and Hispanic neighborhoods as strongly as in white neighborhoods. If we consider good governance as a determinant of SWB, this is in line with previous research that has found diminishing returns of other determinants of SWB, such as income and education, for Black and Hispanic people [28,43].

Interestingly, sensitivity analyses found that individual race/ethnicity did not have a significant moderating effect on the relationship between QoG and life satisfaction. This suggests that while living in a Black/Hispanic neighborhood impacts the relationship between QoG and life satisfaction, actually being a Black or Hispanic individual may not. Logically, this makes sense—a white person living in a Black/Hispanic neighborhood is likely receiving the same quality and timeliness of neighborhood-level public services, such as pothole repair, as a Black/Hispanic person in that neighborhood, and vice versa.

This study is significant for several reasons. First, despite multiple between-nation studies and within-nation studies in Spain and Turkey on the association between local governance and SWB [7,9,10,22], to the authors’ knowledge, there have been no studies on this relationship within the U.S. The U.S. presents a unique sociopolitical context compared to these regions because of its racial and ethnic divisions as well as low trust in government, which have been shown to have significant impacts on many parts of life, including SWB [3,24]. We find that in the U.S. context, local good governance does have a significant and positive impact on SWB, which is in line with previous research from other countries on this relationship [7,9,10]. Second, this study adds to the small but growing literature documenting the diminishing returns of the determinants of SWB on the life satisfaction of Black and Hispanic people in the U.S., also known as the Black and Hispanic SWB paradox [28,43]. We find that in neighborhoods with a higher percentage of Black and Hispanic people, the relationship between local good governance and SWB is less strong than in white neighborhoods.

Future research investigating the relationship between local QoG and SWB in the U.S. could consider more robust composite indicators to model multiple dimensions of QoG rather than a single variable [7,9,10]. Considering the recent electoral successes of local politicians who promise to use government to improve residents’ quality of life, it is important to understand what governments can do to be most effective at this aim, and for whom [44]. Our research suggests that in order to improve the SWB of Black and Hispanic residents, government may need to do more than just respond to basic infrastructure needs.

### Limitations

Our study used the Gallup Daily United States Poll, one of the most robust SWB datasets in the nation. Despite our use of these robust data, the main limitation of our analysis is the spatial resolution of the SWB data available to us: the smallest geographic unit available in the Gallup Daily United States Poll is ZIP code. ZIP codes are not a community-salient or politically salient geography and vary widely in land area and population. SWB data at the census block group or tract level, which are typically smaller and sized to contain similar populations, would allow for better comparison across local geographies. In addition, the most relevant political geographies in Chicago are the 50 aldermanic wards, which do not align with ZIP codes at all (or census tracts or block groups, for that matter) [45]. Considering that we are examining the link between governance and SWB, not having access to SWB data at the geographic level most salient to local governance is a significant limitation of this study. Still, to our knowledge, ZIP code is the smallest geographic unit of analysis available for SWB data in the U.S.

In addition, the geography for the SWB data and pothole data is ZIP code, while that of the neighborhood demographic data is ZCTA. ZIP codes and ZCTAs in Chicago are very similar geographies, but differ slightly—less than 7% area difference. This is because ZIP codes come from the U.S. Postal Service (USPS) for the purpose of organizing mail routes, while ZCTAs were created by the U.S. Census Bureau to approximate ZIP codes but are modified to better suit population analyses [38]. Based on the geographic information available in the SWB data, this slight mismatch is a necessary limitation that is common in ZIP/ZCTA-level analyses [38].

Additionally, the variance accounted for by our models is small, suggesting that neighborhood-level differences in governance account for less than 10% of SWB difference. However, our findings are in line with other papers on SWB with a similar geographic level of analysis using similar covariates and datasets [38,43,46].

The age of the Gallup data is also a limitation in this study. Due to the proprietary nature of the dataset, 2012–2016 are the most recent survey years to which we have access. While we still believe that our results offer insight into the relationship between QoG, SWB, and race, future research should explore these relationships using more recent data.

Finally, 311 data availability limited the scope of our analysis. Of the over one hundred categories of service requests in the City of Chicago 311 dataset, only eleven of the most common had full data available from 2012 to 2016 [12]. Of those eleven, only five had a column in the data table allowing us to ascertain if the service request had been fulfilled: complaints about potholes, rodents, garbage cart maintenance, abandoned vehicles, and tree debris. There were far more pothole complaints than the other four categories, and nearly 87% of pothole complaints were fulfilled, while the city responded to less than 72% of the other four categories [12]. Thus, we decided to focus on pothole complaints for our independent variable. However, if we had a complete 311 dataset, it would be interesting to see how our results change when including all types of 311 service requests.

## 5. Conclusions

The results of this study show that while good local governance benefits SWB in the U.S., its benefits are not distributed equally. People living in Black and Hispanic neighborhoods experience diminishing returns of better local governance compared to people living in white neighborhoods. This reflects a possible additional dimension of the Black and Hispanic SWB paradox, an emerging concept in QoL literature. There are numerous possible reasons for these diminishing returns. For one, in Black and Hispanic neighborhoods in Chicago, which tend to be underinvested and under-resourced compared to white neighborhoods, government maintenance of basic infrastructure could be a lower priority for residents. Other aspects of governance, such as schools, libraries, public transportation, or public safety could be higher priorities and therefore more impactful on SWB.

It could also be that in these neighborhoods, where government has often caused more harm than good, evidenced by the closures of public housing and schools, there is inherent distrust or disregard of government, which negates governance’s impact on SWB [47,48]. Both the questions of priorities and distrust suggest that if the government is intent on using its powers to improve residents’ quality of life, especially in Black and Hispanic neighborhoods, it must do more than just respond to basic needs. Governments may instead need to engage in processes of co-governance and co-production [49] that ensure that residents feel like their voices are heard, that they can trust in government, and that government responds to their most pressing needs in a timely and effective manner. Promising to make government improve residents’ quality of life has become a winning campaign platform [44]; it is important to study how government can actually achieve this goal and do so equitably.

## Figures and Tables

**Table 1:** Descriptive statistics.

Variable	Mean (SD) or Counts (%)	Min	Max
Life satisfaction	7.01 (1.94)	0	10
Median weeks to complete service request	0.89 (0.49)	0.14	1.71
Service requests per ZIP code	4605.32 (2088.02)	337	9340
Age (years)	42.68 (17.16)	18	98
Age-squared	2115.44 (1644.08)	324	9604
Female	2593 (50.1%)	0	1
Non-Hispanic white	2741 (53.0%)	0	1
Non-Hispanic Black	1642 (31.7%)	0	1
Hispanic	790 (15.3%)	0	1
Married	2192 (42.3%)	0	1
Not enough money for food	833 (16.1%)	0	1
Health problems	951 (18.4%)	0	1

**Table 2:** Population-averaged models for the association between service completion time and life satisfaction in Chicago, Illinois, USA, 2012–2016.

Variable	Model 1.1	1.2	1.3
Median weeks to complete service request	−0.04 *	−0.06 ***	−0.05 ***
	(0.07)	(0.07)	(0.06)
Service requests per ZIP code	−0.05 **	−0.05 *	−0.03
	(0.00)	(0.00)	(0.00)
Female		0.05 **	0.06 ***
		(0.06)	(0.06)
Age (years)		−0.27 ***	−0.19 **
		(0.01)	(0.01)
Age-squared		0.30 ***	0.23 **
		(0.00)	(0.00)
Married		0.12 ***	0.09 ***
		(0.05)	(0.05)
Non-Hispanic white		0.04	0.00
		(0.09)	(0.08)
Health problems			−0.12 ***
			(0.08)
Not enough money for food			−0.18 ***
			(0.09)
Model Fit Statistics			
R^2^_marg_	0.004	0.024	0.077
Chi-squared	12.86	118.23	291.49
*p*-value	0.00	0.00	0.00
Observations	5173	5082	5054

Standardized beta coefficients are shown. Robust standard errors are in parentheses. * *p* < 0.05, ** *p* < 0.01, *** *p* < 0.001.

**Table 3:** Neighborhood differences in the association between service completion time and life satisfaction in Chicago, Illinois, USA, 2012–2016.

Variable ^6^	Model 2.1	2.2	2.3	2.4
Interaction effects				
Percent Black/Hispanic population × Weeks to completion ^1^		0.00 *		
		(0.00)		
Above-average Black/Hispanic population × Weeks to completion ^2^				0.27 *
				(0.13)
Main effects				
Percent Black/Hispanic population ^3^	−0.00	−0.01 **		
	(0.00)	(0.00)		
Above-average Black/Hispanic population ^4^			−0.10	−0.33 *
			(0.10)	(0.17)
Weeks to completion ^5^	−0.23 ***	−0.44 ***	−0.23 **	−0.33 ***
	(0.07)	(0.11)	(0.07)	(0.09)
Constant	7.77 ***	7.92 ***	7.71 ***	7.79 ***
	(0.20)	(0.19)	(0.19)	(0.18)
Model Fit Statistics				
R^2^_marg_	0.078	0.079	0.077	0.078
Chi-squared	314.34	343.10	307.85	318.52
Wald Chi-squared		5.30		4.09
*p*-value		0.02		0.04
Observations	5054	5054	5054	5054

Robust standard errors in parentheses. * *p* < 0.05, ** *p* < 0.01, *** *p* < 0.001. ^1^ Relative to ZIP codes with a higher percentage of Black and Hispanic residents and shorter weeks to completion of service requests. ^2^ Relative to ZIP codes with above-average Black and Hispanic populations and shorter weeks to completion of service requests. ^3^ Relative to ZIP codes with a lower percentage of Black and Hispanic residents. ^4^ Relative to ZIP codes with below-average Black and Hispanic populations. ^5^ Relative to shorter weeks to completion of service requests. ^6^ Other variables included but not shown are shown in Table 2.

## Data Availability

City of Chicago 311 pothole reporting data is publicly available online at https://data.cityofchicago.org. The Gallup Daily data presented in this article cannot be made readily available by the authors because the data must be accessed by subscribing to Gallup Analytics. Requests to access the data should be directed to Gallup Analytics at https://www.gallup.com/analytics/213617/gallup-analytics.aspx (accessed on 18 January 2026). Race/ethnicity data is publicly available online at https://data.census.gov in Table DP05, 2016 American Community Survey (ACS) 5-year Demographic and Housing Estimates.

## Abbreviations

The following abbreviations are used in this manuscript:

SWB	Subjective well-being
QoL	Quality of life
QoG	Quality of government
CDOT	Chicago Department of Transportation
OECD	Organization for Economic Cooperation and Development
MDR	Minorities’ Diminished Returns theory
SES	Socioeconomic status
ACS	American Community Survey
GIS	Geographic information system
GEE	Generalized estimating equations
U.S.	United States

## Appendix A. Global Moran’s I Results

**Table A1:** Global Moran’s I statistics for life satisfaction and weeks to completion.

Statistic	Life Satisfaction	Weeks to Completion
Moran’s Index	0.261094	0.872806
Expected Index	−0.017544	−0.017544
Variance	0.006739	0.007009
z-score	3.394337	10.634664
*p*-value	0.000688	0.000000
